# Supplementary material for: Integrative GWAS and co-localisation analysis suggests novel genes associated with age-related multimorbidity
Source: Sci Data. 2023 Sep 25;10:655. doi: 10.1038/s41597-023-02513-4 (PMC10520009; doi:10.1038/s41597-023-02513-4)

Supplementary Information

Table of Contents

SI Table 1 2
SI Table 2 3
SI Table 3 4
SI Figure 1 5
SI Figure 2 6

SI Table 1

For each age-related disease or trait, and for all traits combined ("Total"): the total number of GWAS studies, the number and proportion of these with summary statistics ("with sum stats") available; the minimum, median, and maximum number of individuals included in the GWAS studies; the total number of genome-wide significant trait-associated loci from these GWAS studies ("Total GWAS associations"); and the total number of independent genetic associations after clustering ("Total"), as well as the number of these clusters that included associations from multiple studies ("Replicated") and the number that consisted of just one associated locus ("Unreplicated").

	N GWAS studies		Study Population Sizes				N independent genetic associations (clusters)		
Trait	Total	With sum stats	Min	Max	Median	Total GWAS associations	Replicated	Unreplicated	Total
adiponectin measurement	9	0 (0%)	3,310	67,739	29,347	48	9	19	28
adult onset asthma	7	1 (14%)	327,253	585,812	327,253	336	30	234	264
age-related macular degeneration	16	4 (25%)	2,721	399,047	33,976	108	13	50	63
Alzheimer's disease	62	11 (18%)	2,222	1,462,121	61,571	265	34	85	119
atherosclerosis	15	8 (53%)	31,211	397,126	218,792	142	24	64	88
cartilage thickness measurement	1	0 (0%)	13,013	13,013	13,013	5	0	5	5
chronic kidney disease	24	4 (17%)	4,863	625,219	67,093	92	16	54	70
chronic obstructive pulmonary disease	63	19 (30%)	2,706	517,887	200,766	492	66	132	198
Chronic pain	1	0 (0%)	387,649	387,649	387,649	39	0	39	39
cognitive decline measurement	2	0 (0%)	3,946	3,946	3,946	16	0	16	16
gait measurement	2	0 (0%)	2,946	450,967	450,967	72	0	72	72
glaucoma	37	8 (22%)	4,365	402,223	218,792	518	79	98	177
grip strength measurement	12	2 (17%)	4,608	359,729	359,704	547	138	208	346
healthspan	2	0 (0%)	300,447	837,415	837,415	26	2	22	24
heart disease	146	59 (40%)	2,845	1,030,836	296,525	2,449	315	333	648
heel bone mineral density	12	6 (50%)	114,552	446,000	394,929	3,704	732	567	1,299
hypertension	29	11 (38%)	4,965	517,472	361,141	1,019	181	229	410
idiopathic pulmonary fibrosis	1	0 (0%)	11,259	11,259	11,259	13	0	13	13
lean body mass	24	12 (50%)	2,207	354,808	354,736	7,421	1,045	223	1,268
Lewy body dementia	3	0 (0%)	3,526	3,526	3,526	3	1	0	1
lipid measurement	690	10 (1%)	2,011	859,386	127,326	7,210	682	1,292	1,974
longevity	25	8 (32%)	4,477	837,415	482,000	160	21	38	59
metabolic syndrome	14	0 (0%)	2,554	291,107	291,107	126	13	84	97
obesity	21	8 (38%)	2,928	408,961	204,498	137	26	29	55
osteoarthritis	44	27 (61%)	6,523	613,790	393,873	302	45	64	109
osteoporosis	8	4 (50%)	3,569	407,763	361,141	20	3	12	15
Parkinson's disease	21	2 (10%)	2,755	482,730	417,508	244	47	83	130
rheumatoid arthritis	54	11 (20%)	5,683	369,964	80,799	776	126	231	357
stroke	44	11 (25%)	4,348	922,140	100,321	168	37	55	92
type II diabetes mellitus	90	16 (18%)	4,347	1,407,282	659,316	3,062	484	541	1,025
Total	1394	231 (17%)	2,011	1,462,121	35,634	28,797			

SI Table 2

GeneOntology term mappings for each of the Hallmarks of Ageing, the number of descendant terms in the ontology, and the number of TargetAge genes annotated with each term (including descendent terms) and each Hallmark overall.

Hallmark	GO Term ID	Go Term Name	N descendant terms	N TargetAge Genes	N TargetAge Genes
**Cellular senescence**	GO:0090398	cellular senescence	7	17	17
**Proteostasis**	GO:0006457	protein folding	23	5	70
	GO:0050821	protein stabilization	1	9	
	GO:0030163	protein catabolic process	145	64	
	GO:0010498	proteasomal degradation	51	25	
	GO:0061684	chaperone-mediated autophagy	7	2	
	GO:0061077	chaperone-mediated protein folding	8	2	
**Telomere maintenance**	GO:0000723	telomere maintenance	54	7	7
**Nutrient signalling**	GO:0031667	response to nutrient levels	190	69	72
	GO:0009594	detection of nutrient	2	0	
	GO:0048009	IGF receptor signaling pathway	4	5	
**Mitochondrial genome maintenance**	GO:0000002	mitochondrial genome maintenance	10	0	0
**Epigenetic alteration**	GO:0044728	DNA methylation or demethylation	29	8	58
	GO:0016570	histone modification	215	45	
	GO:0006338	chromatin remodeling	54	24	
**Inflammation**	GO:0006954	inflammatory response	174	65	65
**Immune response**	GO:0006955	immune response	661	92	92

SI Table 3

TargetAge targets with high-quality chemical probes available and no clinical precedence, the name, source, and URL of the probe.

Target ID	Symbol	Probe ID	Source	URL
ENSG00000119778	ATAD2B	BAY-850	Bromodomains chemical toolbox	https://www.nature.com/articles/s41467-019-09672-2#article-info
			Chemical Probes.org (legacy)	https://new.chemicalprobes.org/?q=BAY-850
			SGC Probes	http://www.thesgc.org/chemical-probes/BAY-850
		GSK8814	Bromodomains chemical toolbox	https://www.nature.com/articles/s41467-019-09672-2#article-info
			SGC Probes	http://www.thesgc.org/chemical-probes/GSK8814
			Chemical Probes.org (legacy)	https://new.chemicalprobes.org/?q=GSK8814
ENSG00000171634	BPTF	NVS-BPTF-1	SGC Probes	http://www.thesgc.org/chemical-probes/NVS-BPTF-1
		TP-238	SGC Probes	http://www.thesgc.org/chemical-probes/TP-238
ENSG00000104885	DOT1L	canSAR828356	Chemical Probes.org (legacy)	https://new.chemicalprobes.org/?q=canSAR828356
			Protein methyltransferases chemical toolbox	https://www.nature.com/articles/s41467-018-07905-4
		canSAR851884	Chemical Probes.org (legacy)	https://new.chemicalprobes.org/?q=canSAR851884
			Probe Miner	http://probeminer.icr.ac.uk/#/Q8TEK3
		canSAR856426	Chemical Probes.org (legacy)	https://new.chemicalprobes.org/?q=canSAR856426
			Probe Miner	http://probeminer.icr.ac.uk/#/Q8TEK3
ENSG00000198945	L3MBTL3	UNC1215	Nature Chemical Biology Probes	http://www.nature.com/nchembio/chemical_probes.html
			SGC Probes	http://www.thesgc.org/chemical-probes/UNC1215
			Chemical Probes.org (legacy)	https://new.chemicalprobes.org/?q=UNC1215
ENSG00000168906	MAT2A	AG-270	Chemical Probes.org (legacy)	https://new.chemicalprobes.org/?q=AG-270
ENSG00000078142	PIK3C3	SAR405	Nature Chemical Biology Probes	http://www.nature.com/nchembio/chemical_probes.html
ENSG00000198890	PRMT6	SGC6870	SGC Probes	http://www.thesgc.org/chemical-probes/SGC6870
		canSAR1301406	Protein methyltransferases chemical toolbox	https://www.nature.com/articles/s41467-018-07905-4
			Chemical Probes.org (legacy)	https://new.chemicalprobes.org/?q=canSAR1301406
ENSG00000142178	SIK1	MRIA9	SGC Probes	http://www.thesgc.org/chemical-probes/MRIA9
		canSAR2945471	Chemical Probes.org (legacy)	https://new.chemicalprobes.org/?q=canSAR2945471
ENSG00000178950	GAK	CA93.0	SGC Probes	http://www.thesgc.org/chemical-probes/CA93.0
ENSG00000137764	MAP2K5	canSAR326840	Chemical Probes.org (legacy)	https://new.chemicalprobes.org/?q=canSAR326840
ENSG00000160584	SIK3	MRIA9	SGC Probes	http://www.thesgc.org/chemical-probes/MRIA9
		canSAR2945471	Chemical Probes.org (legacy)	https://new.chemicalprobes.org/?q=canSAR2945471

SI Figure 1

The effect of using a more stringent overlap criteria on the number of clusters and sub-cluster communities in the graph. The minimum overlap between the tag variants of two genetic associations, defined using the Jaccard Index (i.e. the ratio of the intersection over the union), required to define an edge between two genetic associations was varied from >0 to 1, to compare the number of a) clusters and b) sub-cluster communities identified in the resulting graphs.

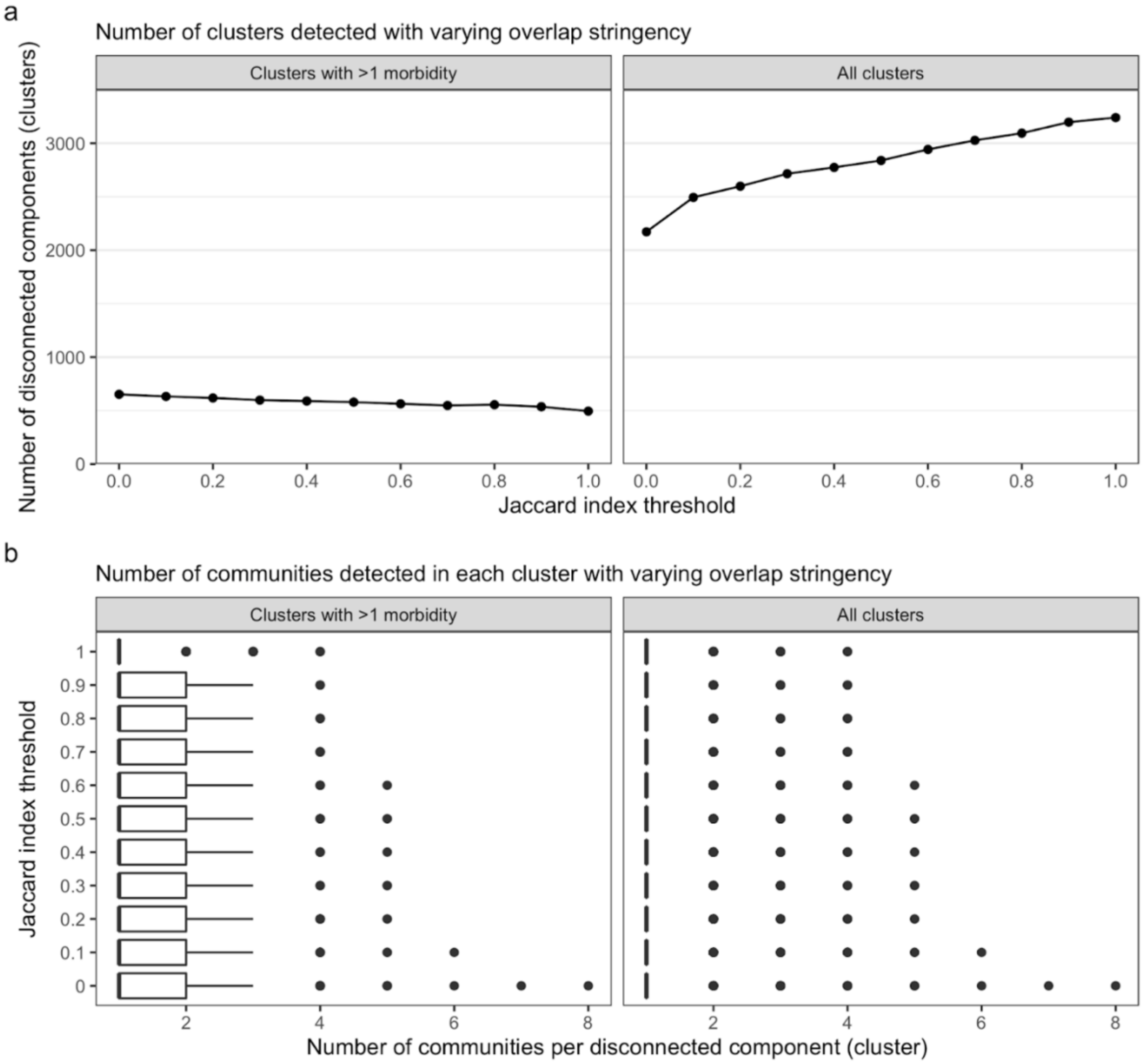

SI Figure 2

The number and proportion of independent genetic associations shared between age-related diseases and traits. Proportions are relative to the total number of independent genetic associations identified for the trait on the horizontal axis; darker blue indicates a greater proportion of shared genetic associations. Cells marked with an asterisk are statistically significant after adjusting for multiple testing of all pairwise combinations of traits. Traits are ordered by the total number of implicated genetic association clusters.

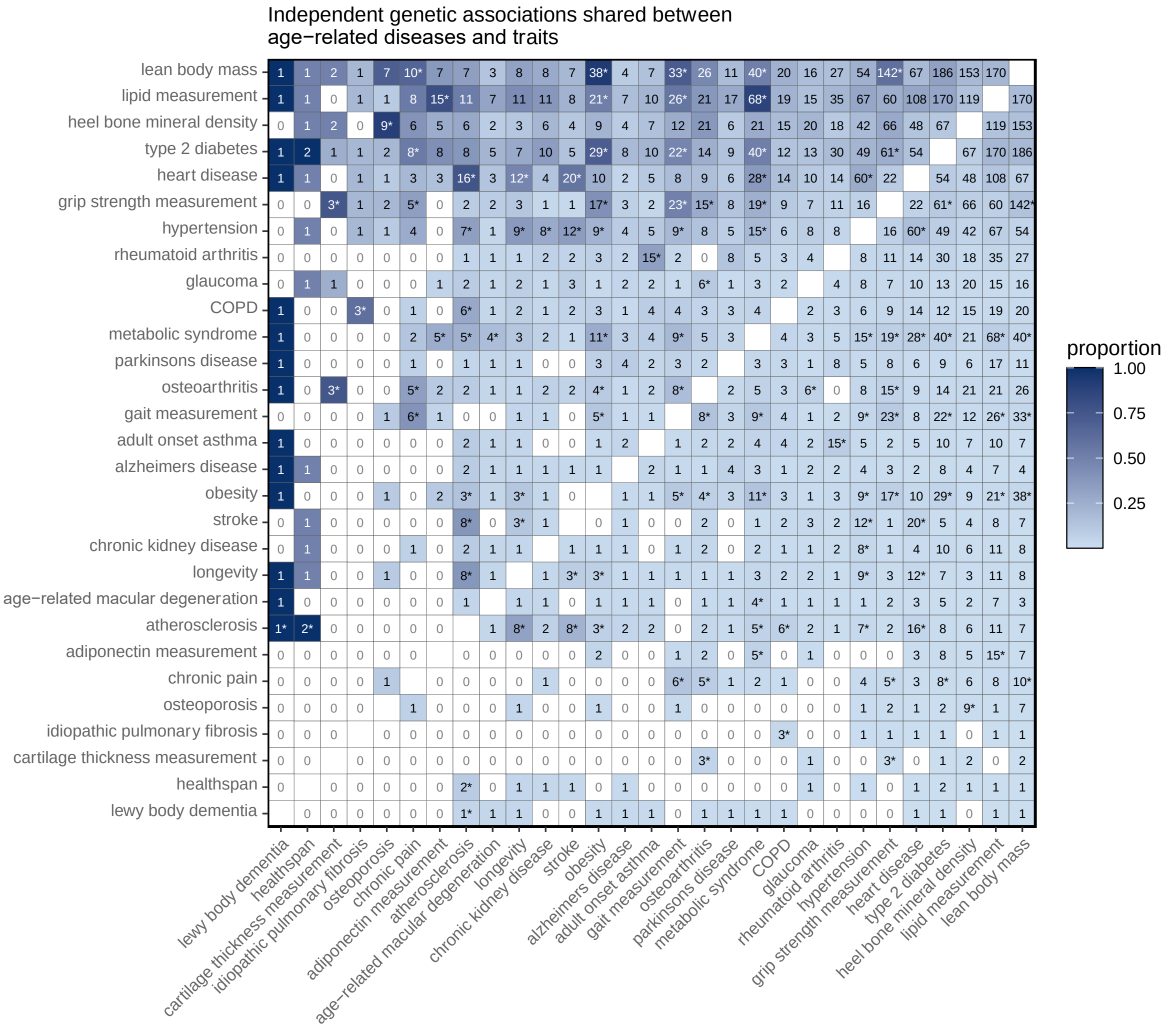

Supplement: Supplementary file 1 — Supplementary Information [file 41597_2023_2513_MOESM1_ESM.pdf]
